# The Role of GPR39 in Regulating Osteoblast Function, Bone Matrix Quality, and Gender‐Specific Bone Homeostasis

**DOI:** 10.1002/jcp.70095

**Published:** 2025-10-03

**Authors:** Biplab Chaterjee, Gal Gozlan, Chen Abramovitch‐Dahan, Anton Davydok, Anat Reiner‐Benaim, Johannes Krug, Katharina Jähn‐Rickert, Björn Busse, Noam Levaot

**Affiliations:** ^1^ Department of Physiology and Cell Biology Ben‐Gurion University of the Negev Beer‐Sheva Israel; ^2^ Institute of Material Physics, Hereon Outstation at DESY Helmholtz Zentrum Hereon Hamburg Germany; ^3^ Department of Epidemiology, Biostatistics and Community Health Sciences Ben‐Gurion University of the Negev Beer‐Sheva Israel; ^4^ Department of Osteology and Biomechanics University Medical Center Hamburg‐Eppendorf Hamburg Germany; ^5^ Interdisciplinary Competence Center for Interface Research Hamburg Germany

**Keywords:** bone composition, bone mineralization, Col1a2, collagen, collagen deposition, osteoblasts, osteoporosis, ovariectomy, zinc, ZnR/GPR39

## Abstract

GPR39, a zinc‐sensing receptor, is essential for bone homeostasis in male mice through regulation of osteoblast function and matrix composition. This study examined the effects of GPR39 deficiency in female mice using both global and osteoblast lineage‐specific GPR39 knockout models (*Gpr39* ^*Ob*−*/Ob*−^). In vivo, GPR39‐deficient female mice exhibited reduced bone mass, increased mineralization rates, and significantly lower and more variable serum levels of pro‐collagen type I N‐propeptide (PINP), indicating impaired collagen synthesis and matrix remodeling. OVX models further demonstrated that GPR39 deficiency exacerbates estrogen‐deficiency‐induced bone loss, highlighting its protective role in postmenopausal‐like states. Osteoblast lineage‐specific GPR39 deletion replicated the skeletal abnormalities observed in global knockouts, revealing that GPR39 activity in the osteoblast lineage is indispensable for proper collagen deposition and mineralization. Western blot analysis of *Gpr39* ^*Ob*−*/Ob*−^ osteoblasts confirmed reduced extracellular collagen levels, while quantitative mRNA analysis of Col1a2 revealed zinc signaling through GPR39 as a key regulator of collagen production. Zinc‐induced Col1a2 expression, dependent on GPR39 and mediated via Gα_q_ signaling, was abolished in GPR39‐deficient osteoblasts. These findings provide insights into how zinc signaling via GPR39 regulates osteoblast function and collagen synthesis, emphasizing its role in maintaining matrix composition. Targeting GPR39 may offer novel therapeutic strategies for osteoporosis and other bone disorders characterized by impaired matrix remodeling.

## 1 Introduction

The capacity of bone to endure the substantial loads imposed upon it throughout an individual's lifespan is attributed to its distinctive matrix composition and the continuous turnover of this matrix, mediated by the coordinated activity of bone cells (Hadjidakis and Androulakis 2006; Sims and Vrahnas 2014). The bone matrix consists of mineralized carbonated hydroxyapatite, type I collagen, and water, alongside minor constituents including other collagen types, noncollagenous proteins, and proteoglycans (Jablonski 1990). Alterations in bone matrix composition, such as an elevated mineral‐to‐matrix ratio, are commonly observed in bone pathologies and may correlate with an increased risk of fracture. In osteoporosis, these compositional changes arise due to a diminished rate of bone formation, whereas in osteogenesis imperfecta, they result from disrupted collagen processing by osteoblasts (Gourion‐Arsiquaud et al. 2009; Isaksson et al. 2010; Shapiro et al. 2013). Altered bone composition may also result from inadequate nutrition. Given that calcium constitutes a primary component of hydroxyapatite crystals in bone, a deficiency in dietary calcium—coupled with insufficient vitamin D, which is critical for calcium absorption—can lead to diminished bone mineral content (BMC) and a consequent reduction in bone matrix stiffness (Rodríguez‐Martínez and García‐Cohen 2002). Another nutritional element that is important for bone health is zinc. Zinc is a vital trace element that plays a significant role in various biological processes, including bone development, maintenance, and repair, as well as hormonal regulation (Levaot and Hershfinkel 2018). Zinc is crucial for this balance and overall bone health; its deficiency is associated with growth arrest in children and bone loss in adults, particularly in postmenopausal women (Hyun et al. 2004; Karaaslan et al. 2014; Prasad et al. 1961; Yamaguchi 2010; Zheng et al. 2014). Moreover, zinc interacts with several hormones that influence bone metabolism. It is essential for insulin synthesis and secretion, thyroid hormone metabolism, and the synthesis of sex hormones like testosterone and estrogen. Zinc's role as a cofactor for enzymes involved in these hormonal pathways highlights its broad impact on endocrine function and bone homeostasis (Baltaci et al. 2019; Hyun et al. 2004; Severo et al. 2019). Although zinc supplementation has been shown to enhance bone formation and collagen production, the precise molecular mechanisms by which zinc regulates these processes remain incompletely understood.

G protein‐coupled receptor 39 (GPR39) has been identified as a zinc‐sensing receptor (ZnR) that responds to extracellular zinc levels and regulates a variety of zinc‐mediated processes, including hormonal regulation. GPR39 is expressed in pancreatic beta cells, where it is involved in zinc‐induced insulin secretion and glucose homeostasis (Hershfinkel et al. 2007; Storjohann et al. 2008; Yasuda et al. 2007). It has also been implicated in the modulation of gastrointestinal hormones such as ghrelin, which influences appetite control and energy metabolism (Popovics and Stewart 2011).

Our previous research established a foundational role for GPR39 in bone matrix composition and strength, particularly in male mice. We demonstrated that GPR39‐deficient (Gpr39^−/−^) male mice exhibit a significant increase in the bone mineral‐to‐matrix ratio, primarily due to a marked reduction in collagen content. This imbalance resulted in bones with higher mineralization but compromised mechanical properties, making them more brittle. Importantly, these mice showed an increase in the number of osteoblasts without corresponding changes in osteoclast distribution, suggesting that while osteoblast activity was enhanced, the quality of the bone matrix was impaired due to deficient collagen synthesis. Further analysis revealed that GPR39 influences osteoblast function by modulating collagen production, a key component of the bone extracellular matrix critical for maintaining bone strength and resilience (Jovanovic et al. 2018).

Building on these findings, the current study investigates the role of GPR39 in bone homeostasis in female mice, who have a distinct hormonal environment and are particularly susceptible to bone loss under estrogen‐deficient conditions, such as menopause. Using the ovariectomy (OVX) method to model estrogen deficiency, we examine the impact of GPR39 deficiency in both female and OVX mice to better understand how this receptor mediates the effects of hormonal changes on bone metabolism. Given the broad impact of hormonal regulation on various bone cell types and their functions, we further dissect the cellular source of GPR39's effects on the skeleton by employing a tissue‐specific deletion approach targeting the osteoblast lineage. This allows us to directly assess the contribution of osteoblast‐expressed GPR39 to bone matrix composition, strength, and overall bone quality.

Our findings reveal that osteoblast lineage‐specific deletion of GPR39 replicates the skeletal phenotype observed in germline GPR39‐deficient mice, highlighting that GPR39 activity within osteoblasts is crucial for maintaining bone strength and quality, especially under conditions of hormonal deficiency. These insights provide a deeper understanding of the interplay between zinc signaling, GPR39 activity, and hormonal regulation in bone biology, suggesting that targeting GPR39 could offer novel therapeutic strategies for bone disorders characterized by impaired matrix composition and remodeling, such as osteoporosis.

## 2 Materials and Methods

### 2.1 Animals and Experimental Design

Female Gpr39 knockout (*Gpr39* ^−/−^) (Moechars et al. 2006) and wild‐type (WT) mice on a C57BL/6J background were utilized. Additionally, osteoblast lineage‐specific GPR39 knockout mice (*Gpr39* ^*Ob*−*/Ob*−^) were generated by crossing Gpr39 floxed mice (*Gpr39* ^*fl/fl*^) with Osx1‐GFP::Cre transgenic mice (Jackson Laboratory, Strain 006361), expressing Cre recombinase under the Osterix (Osx) promoter. In the absence of doxycycline, *Gpr39* ^*Ob*−*/Ob*−^ mice exhibited targeted deletion of GPR39 in osteoblasts and their progeny. Mice were maintained under standard laboratory conditions (12‐h light/dark cycle) with ad libitum access to water and standard rodent chow.

To label mineralizing bone surfaces, calcein (10 mg/kg; Sigma Aldrich, St. Louis, MO, USA) was administered via subcutaneous injection 4 days and 1 day prior to sacrifice, enabling quantification of bone formation rates. All procedures adhered to ethical guidelines approved by the Institutional Animal Care and Use Committee (IACUC).

### 2.2 Generation of Mice With Floxed GPR39 Allele

The *Gpr39* ^*fl/fl*^ mice were generated using CRISPR‐Cas9‐mediated gene targeting (Yang et al. 2014). The insertion of loxP recombination sites was performed in two stages. First, a single loxP site (LoxUP) was inserted 750 bp upstream of the GPR39 transcriptional start site in a nonconserved region to minimize transcriptional interference. Subsequently, a second loxP site (LoxDown) was inserted 1603 bp downstream, 400 bases from the end of exon 1, resulting in mice with flanking loxP sites.

The mouse strain was created using a modified CRISPR‐Cas9 protocol. T7‐sgRNA templates were in vitro transcribed using the MEGAshortscript T7 kit (Thermo Fisher Scientific), and Cas9 mRNA was synthesized with the mMESSAGE mMACHINE T7 kit (Thermo Fisher Scientific) from a PCR‐amplified px330 plasmid template encoding the humanized Streptococcus pyogenes Cas9. Zygotes from C57BL/6J embryos were injected with a mix of Cas9 mRNA (100 ng/µL) and sgRNA (50 ng/µL) in injection buffer (10 mM Tris, pH 7.5; 0.1 mM EDTA) following standard procedures approved by Ben‐Gurion University. ICR (CD‐1) female mice served as foster mothers. F0 mice were genotyped via PCR to confirm loxP site insertion (see sgRNA and primer sequences below).

### 2.3 Primer and Oligonucleotides

sgRNA targeting sequences: for loxUP insertion, AATCCCTCCACCCGTTTAAG; for loxDown insertion, CAGAGAGCCTTAACTCGGCA.

Genotyping: LoxUP, forward primer, TGCATATGTCCACTCACTTTCTTTC; reverse primer, AGCTCATGGGCTCTCAAAGAGGT; LoxDown, forward primer, GACAGGGACAAGTCCTTCAGAGA; reverse primer, GTATCCCTGAGAAGCCAGTTTCTTCC; Osterix cre, forward primer: GAGAATAGGAACTTCGGAATAGTAAC; reverse primer, CCCTGGAAGTGACTAGCATTG.

Deletion validation primers: forward primer, AGAATCCCTCCACCCGTTTG; reverse primer, GGCCCAGCAGAAATCTAAGC.

### 2.4 Ovariectomy (OVX) Experiments

To elucidate the role of GPR39 in bone homeostasis under estrogen‐deficient conditions, ovariectomy (OVX) or sham surgeries were performed on *Gpr39* ^−*/*−^ and WT mice at 8 and 12 weeks of age. Mice were anesthetized with ketamine (100 mg/kg) and xylazine (10 mg/kg). For OVX, a dorsolateral incision was made, and the ovaries were removed to induce estrogen deficiency, while sham‐operated mice underwent the same procedure without ovary removal. Postoperative monitoring included daily recovery checks and pain management as needed. Four weeks postsurgery, mice were sacrificed, and uterus weight was measured to confirm OVX effectiveness, indicated by significantly reduced uterus weight compared to sham controls.

### 2.5 Microcomputed Tomography

Microcomputed tomography (µCT) analysis of L5 vertebrae from 12‐ and 16‐week‐old female mice was performed using a SkyScan 1174v2 scanner. Scans were acquired at a voxel size of 7.6 µm, with 50 kV source voltage, 800 µA current, a 0.25 mm aluminum filter, 5000 ms exposure time, 0.6° rotation steps, and frame averaging of 2. A global thresholding method was applied for image segmentation.

Trabecular architecture was assessed within a region of interest (ROI) normalized to bone length, positioned between sections below and above the end plates. Analysis was conducted using CTAn software (Bruker).

### 2.6 Dynamic Histomorphometry

Dynamic histomorphometry was performed on L4 vertebrae. Bones were fixed in 4% paraformaldehyde (0.1 M phosphate buffer) and embedded in methyl methacrylate. Sections were cut at 4 µm using a Leica RM2255 microtome. Trabecular regions were imaged at 20× magnification using an Olympus IX81 microscope with a CoolSNAP HQ2 camera and CellSens Dimension software. Two sections per mouse were analyzed using ImageJ2 software.

### 2.7 Tartrate‐Resistant Acid Phosphatase (TRAP) Staining

L3 vertebrae were fixed in 4% paraformaldehyde (0.1 M phosphate buffer), decalcified in 10% EDTA, and embedded in paraffin. In total, 6 µm sections were stained for TRAP using a kit (Sigma‐Aldrich) following the manufacturer's protocol.

### 2.8 Primary Osteoblast Cell Isolation Culture

Primary osteoblasts were isolated from long bones by dissecting, removing bone marrow, and cutting bones into 1–2 mm fragments. Fragments underwent five sequential collagenase and EDTA digestions with HBSS washes between steps. The final cell pellet was resuspended in growth medium (α‐MEM with 10% FBS, 1% penicillin–streptomycin, 1% glutamine, and 100 µg/mL ascorbate) and cultured under hypoxic conditions for four passages. At passage five, cells were plated for experiments.

Osteoblast differentiation was induced by culturing cells in osteogenic medium (α‐MEM with 10% FBS, 1% penicillin–streptomycin, 1% glutamine, 100 µg/mL ascorbate, and 4 mM β‐glycerophosphate) in a standard CO_2_ incubator with medium changes every 2 days.

### 2.9 Extracellular Matrix Fractionation

Primary osteoblasts were cultured under differentiation conditions. On Days 14, 21, and 28, cells were processed for collagen fraction analysis using the method by Graham et al. (2010).

### 2.10 ELISA Assays

Serum PINP levels were quantified in 16‐week‐old wild‐type and *Gpr39* ^−/−^ mice using the IDS Rat/Mouse PINP EIA kit according to the manufacturer protocol. Serum TNFRSF11B (osteoprotegrin, OPG) and TRANCE/TNFSF11 (Receptor Activator of Nuclear factor kappa‐Β Ligand, RANKL) levels were quantified using Abcam SimpleStep ELISA kits (ab203365 and ab269553, respectively) according to manufacturer protocols.

### 2.11 Statistical Analysis

Data are presented as means ± standard deviation. Statistical significance was determined using an unpaired Student's *t*‐test for comparisons between two groups or one‐way ANOVA for multiple group comparisons. Two‐way ANOVA including interaction effect was used to assess zinc and strain differences and post hoc analysis was used added for pairwise comparisons among all subgroups. A *p* < 0.05 was considered statistically significant.

### 2.12 Generative Artificial Intelligence Tools

ChatGPT4o was used only to assist in language editing.

## 3 Results

### 3.1 The Role of GPR39 in Regulation of Bone Homeostasis in Female Mice

**Figure 1:**
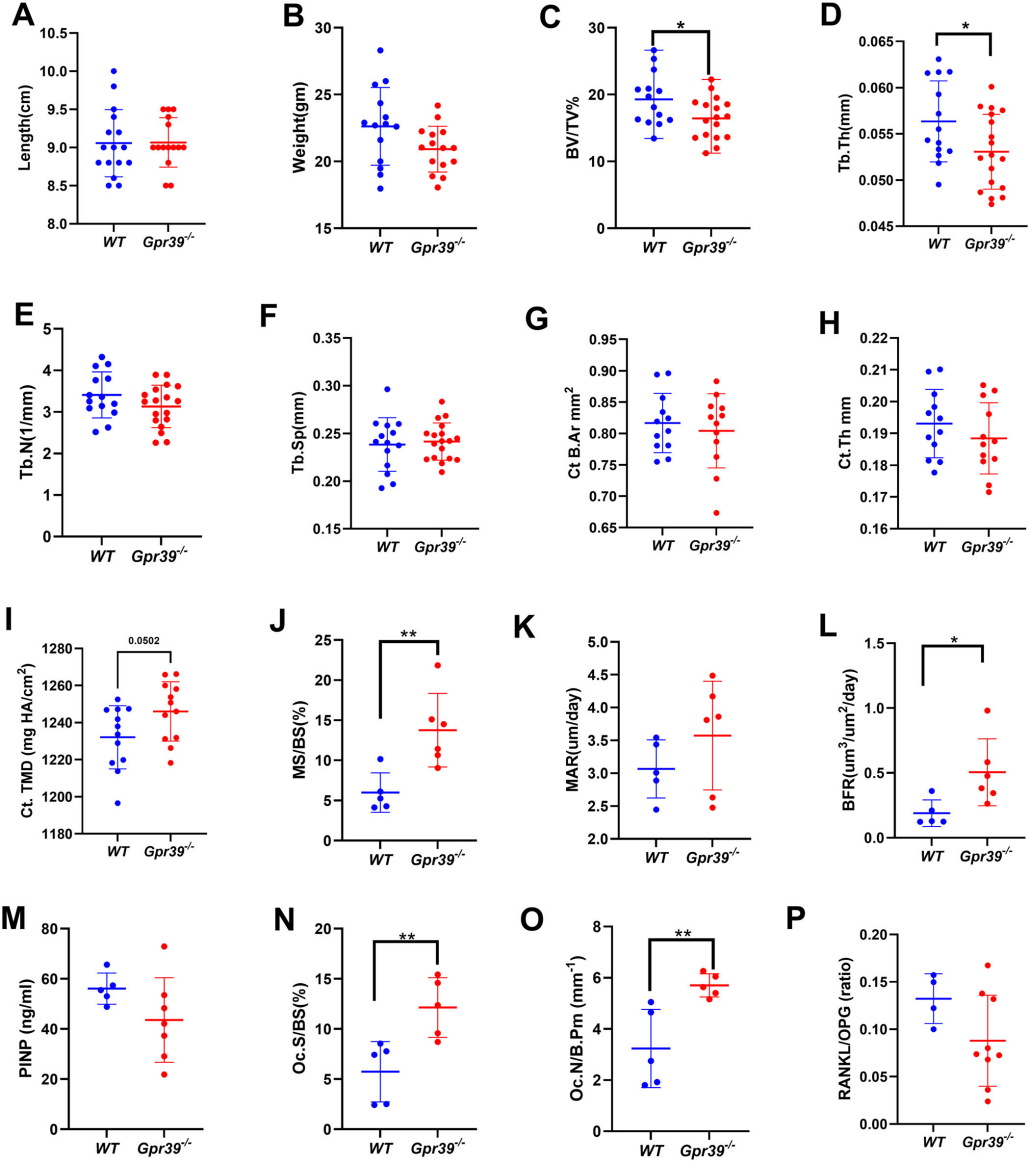
The effects of GPR39 deficiency on bone structure and metabolism in female mice. Sixteen‐week‐old *Gpr39* ^−/−^ and WT mice were compared. (A) Body length and (B) body weight. µCT derived structural analysis of vertebral (C) trabecular bone volume to total volume ratio (BV/TV), (D) trabecular thickness (Tb.Th); (E) trabecular number (Tb.N); (F) trabecular separation (Tb.Sp); femural (G) cortical bone area (Ct B. Ar); (H) cortical thickness (Ct.Th), and (I) cortical mean mineral content (Ct. TMD). Dynamic histomorphometric analyses of (J) mineralizing surface per bone surface (MS/BS), (K) mineral apposition rate (MAR), and (L) bone formation rate (BFR) in L4 vertebrae; (M) serum PINP levels; (N) osteoclast surface per bone surface (Oc.S/B.S); (O) osteoclast number per bone perimeter (Oc.N/B.pm); (P) the ratio between serum RANKL and OPG. Data presented as means ± SD (unpaired Student's *t*‐test), with sample sizes of *n* = 14 (WT) and *n* = 18 (*Gpr39* ^−/−^) for (A–I) and *n* = 5 (WT) and *n* = 5–7 (*Gpr39* ^−/−^) for (J–O). *p < 0.05 and **p < 0.01.﻿﻿

To determine the effects of GPR39 deletion on the skeleton of female mice, we first assessed whether GPR39 deficiency led to any metabolic or growth disturbances by comparing the body weight and length of female *Gpr39* ^−*/*−^ mice to wild‐type (WT) controls. At 4 months of age, *Gpr39* ^−*/*−^ females had normal body length (Figure 1A) and weight (Figure 1B), indicating no significant metabolic or growth abnormalities.

To examine how GPR39 deficiency affects bone structural parameters in mature female mice, we performed microCT analysis on the vertebral and femural bones. Gpr39^−/−^ females exhibited a 14% reduction in vertebral trabecular bone volume fraction compared to WT mice (Figure 1C). Analysis of trabecular architecture showed a 5.81% decrease in trabecular thickness; however, there were no significant differences in trabecular number or trabecular separation (Figure 1D–F). Femoral cortical analysis showed no significant bone structural changes (Figure 1G,H). However, Gpr39^−/−^ females exhibited a near significant higher BMC. These findings suggest that GPR39 regulates vertebral bone structure and femoral bone composition.

Next, we investigated whether changes in bone turnover by bone cells could account for the observed structural alterations in Gpr39^−/−^ female mice. Histomorphometric analysis of the L4 vertebral trabecular bone showed significantly higher mineralizing surfaces in Gpr39^−/−^ females compared to WT (Figure 1J). However, the mineral apposition rate was not significantly different between the groups (Figure 1K). Consequently, bone formation rate, which integrates both mineralizing surface and mineral apposition rate, was higher in 4‐month‐old Gpr39^−/−^ females (Figure 1L).

To assess the impact of GPR39 deficiency on collagen matrix formation in vivo, we compared serum levels of pro‐collagen type I N‐propeptide (PINP) between Gpr39^−/−^ and WT female mice. Although the average serum PINP levels did not differ significantly between the groups, the variance was higher in Gpr39^−/−^ females, approaching significance (*p* = 0.07), suggesting more variability in collagen processing (Figure 1M). These results point for abnormal osteoblast function in Gpr39^−/−^ females, characterized by an increased mineralization rate and variability in collagen processing.

To assess whether enhanced osteoclastic activity contributes to the osteoporotic phenotype observed in Gpr39^−/−^ females, osteoclast distribution within vertebral bone compartments was examined. Histological evaluation of tartrate‐resistant acid phosphatase (TRAP)‐stained sections demonstrated a two‐fold increase in osteoclast‐covered bone surfaces in Gpr39^−/−^ specimens relative to wild‐type controls (Figure 1N). Moreover, the number of osteoclasts per bone surface was elevated by 76.2% in Gpr39^−/−^ females when compared with wild‐type counterparts (Figure 1O). To elucidate potential mechanisms underlying this augmented osteoclast distribution, systemic levels of the osteoclastogenic cytokine, RANKL, and its decoy receptor OPG were quantified. Serum concentrations of RANKL and OPG did not differ significantly between genotypes (Figure 1P), indicating that GPR39 likely governs osteoclast localization predominantly through local regulatory pathways rather than systemic modulation. Collectively, these findings support the conclusion that reduced bone mass in Gpr39^−/−^ females arises, at least in part, from excessive osteoclast presence on bone surfaces.

### 3.2 GPR39 Deficiency Increases Susceptibility to Bone Loss Induced by Sex Hormone Deprivation

**Figure 2:**
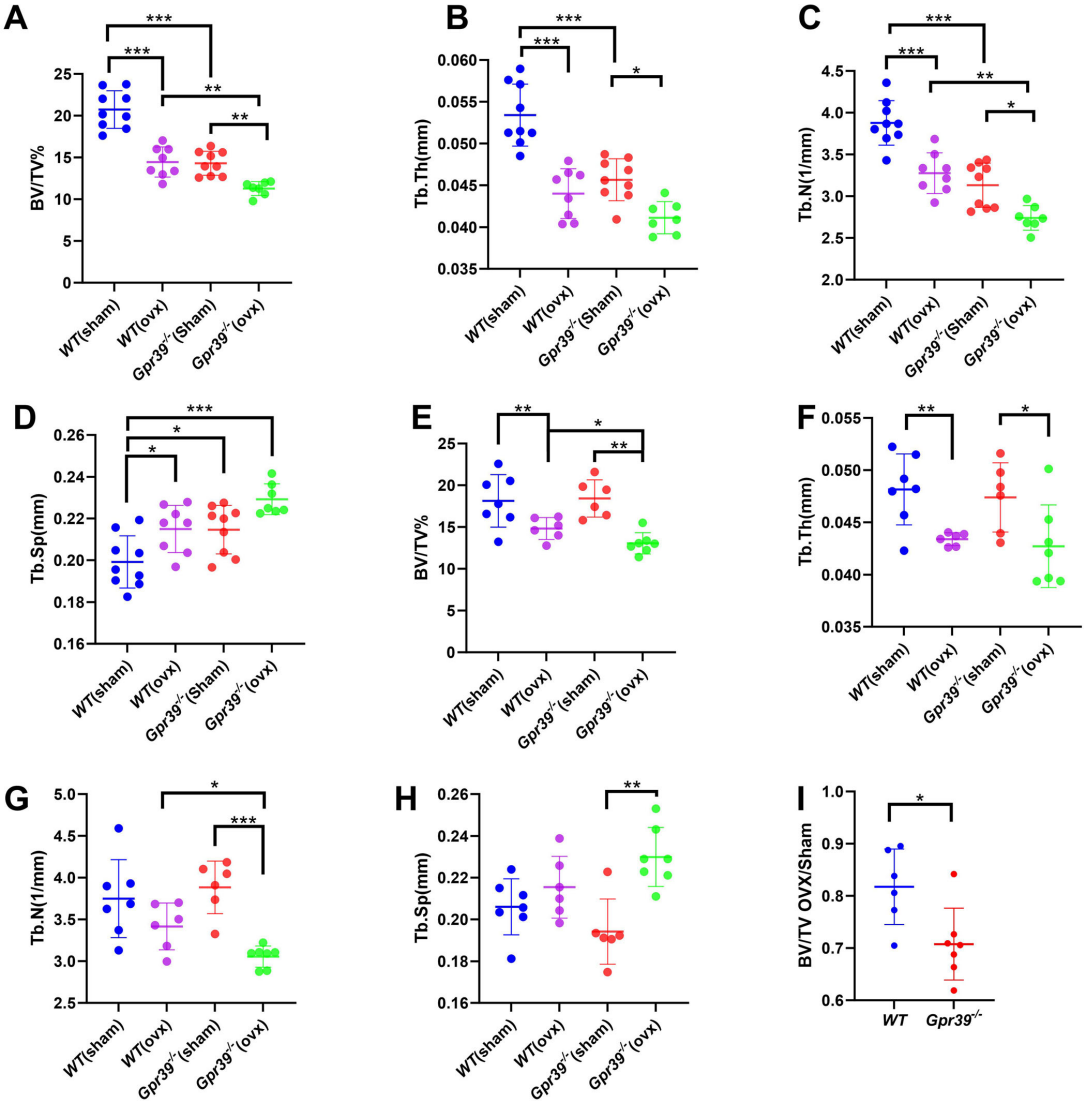
GPR39 deficiency aggravates ovariectomy‐induced bone loss. Morphometric analyses (μCT) of vertebrae from ovariectomized (OVX) mice. The effects of sex hormone deprivation were tested in two age groups of skeletally mature mice (16 weeks old; A–D) where baseline bone volume fraction is lower, and developing mice (12 weeks old; E–I) where baseline bone volume fraction is similar between WT and *Gpr39* ^*−/−*^ females. (I) Percentage reduction of bone mass post‐OVX. Data were divided by the average of the corresponding sham‐operated group. Data presented as means ± SD (one‐way ANOVA, A–H and unpaired Student's *t*‐test, I). *p < 0.05, **p < 0.01, and ***p < 0.001.

Given that GPR39 deficiency is associated with higher osteoclast activity, we further investigated whether GPR39 functions to maintain bone homeostasis by restraining osteoclast activity, particularly under conditions of sex hormone deprivation. To test this hypothesis, initially, we performed ovariectomy on skeletally mature (16‐week‐old) WT and *Gpr39* ^−/−^ mice. As expected, OVX significantly reduced vertebral bone volume fraction in WT mice compared to their sham‐operated controls (Figure 2A). Interestingly, the baseline bone volume fraction in sham‐operated *Gpr39* ^−/−^ females was similar to that of OVX WT mice, highlighting their already osteoporotic phenotype. OVX further reduced bone volume fraction in *Gpr39* ^−/−^ females compared to sham‐operated *Gpr39* ^−/−^ mice (Figure 2A). Analysis of trabecular architecture showed that OVX *Gpr39* ^−/−^ mice had the lowest trabecular thickness and number among all groups (Figure 2B,C), while trabecular separation was highest (Figure 2D). Notably, the lower bone volume structure in sham‐operated *Gpr39* ^−/−^ females complicated the interpretation of OVX‐specific effects. Therefore, it was challenging to determine if *Gpr39* ^−/−^ females are inherently more susceptible to OVX‐induced bone loss. To clarify these findings, we repeated the experiment on younger, developing mice (8 weeks old), which display a similar baseline vertebral trabecular bone volume fraction. Females from both WT and *Gpr39* ^−/−^ groups underwent either OVX or a sham operation, and bone parameters were evaluated 4 weeks later, at 12 weeks of age. As expected, at this age, µCT analysis showed no significant difference in bone mass or other structural parameters between sham‐operated *Gpr39* ^−/−^ and WT mice. However, OVX *Gpr39* ^−/−^ mice had a more pronounced reduction in bone mass and trabecular number compared to OVX WT mice (Figure 2E,G), while trabecular thickness and separation remained unchanged (Figure 2F,H). Assessment of ovariectomy‐induced bone loss showed significant higher percentage of reduction in *Gpr39* ^−/−^ females indicating that ZnR/GPR39 deficiency increases susceptibility to OVX‐induced bone loss in developing mice (Figure 2I).

### 3.3 GPR39 Function in the Osteoblast Lineage Regulates Bone Homeostasis

**Figure 3:**
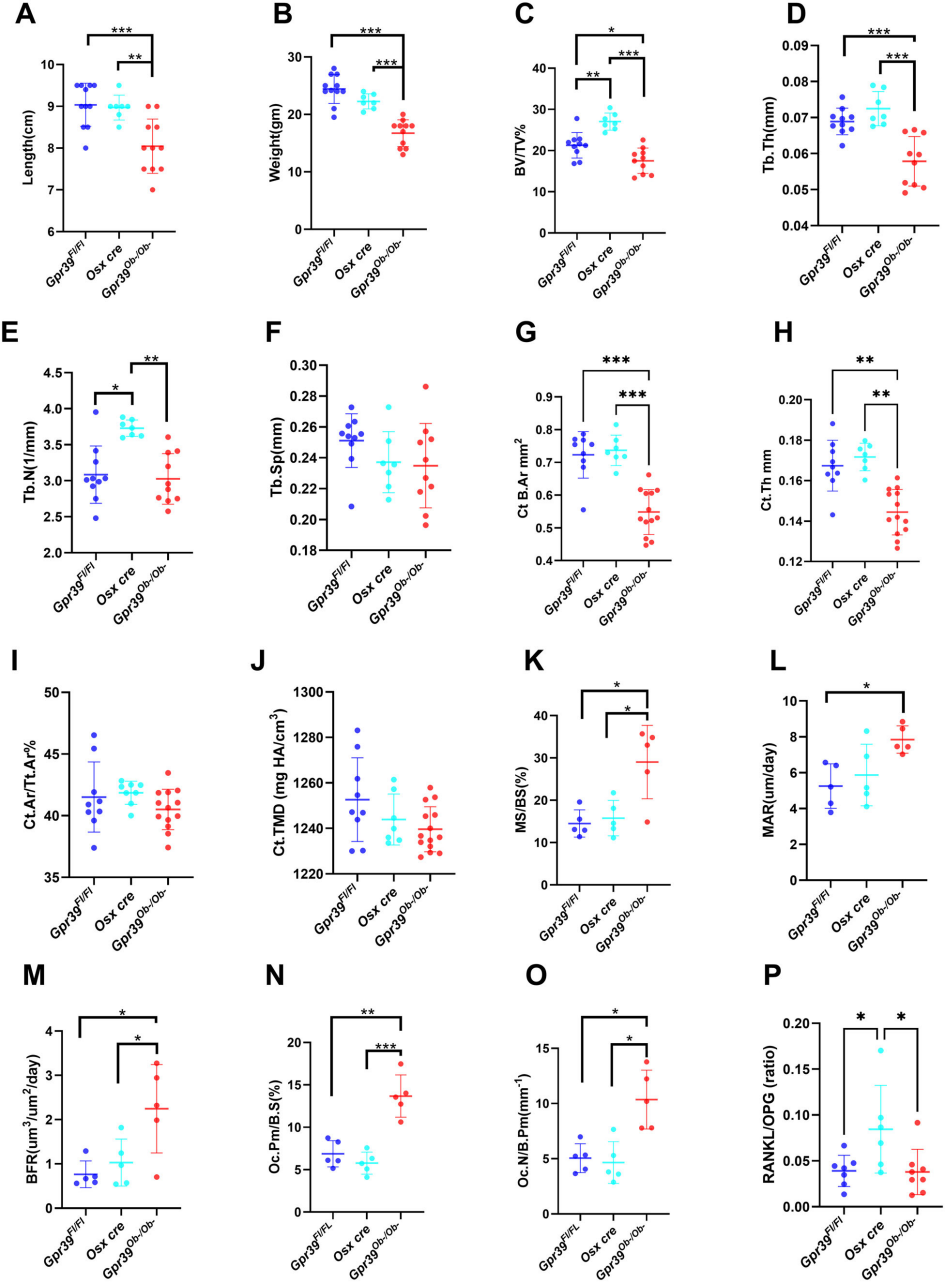
The effects of osteoblast lineage‐specific deletion of GPR39 on bone structure and metabolism in female mice**.** Sixteen‐week‐old female mice with a conditional deletion of GPR39 in the osteoblast lineage (Gpr39^Ob−/Ob−^) were compared to mice harboring the floxed allele (Gpr39^Fl/Fl^) and osterix cre expressing mice (Osx cre). (A) Body length; (B) body weight; (C–F) μCT analysis of vertebral trabecular bone structural parameters. Femural (G) cortical bone area (Ct B. Ar); (H) cortical thickness (Ct.Th); (I) cortical bone area to total area (Ct.Ar/Tt.Ar); (J) cortical mean mineral content (Ct. TMD); (K–O) histomorphometric analyses of vertebral bone sections, and (P) the ratio between the serum concentrations of RANKL and OPG. Data presented as means ± SD (one‐way ANOVA). *p < 0.05, **p < 0.01, and ***p < 0.001.

To elucidate the cellular mechanisms underlying GPR39 regulation of bone homeostasis, we generated a mouse in which the first exon of GPR39 was flanked by LoxP recombination sites (*Gpr39* ^*Fl/Fl*^) (Figure S1A). To specifically knockout GPR39, these mice were crossed with Cre recombinase‐expressing mice under the control of the osterix promoter (Gpr39^Ob−/Ob−^). In our experience, GPR39 expression is low and despite several attempts we could not detect its mRNA or protein in bones in vivo limiting the assessment of deletion efficiency. To confirm the specific deletion of GPR39 in the osteoblast lineage, tibiae (an osteocyte‐rich tissue) were isolated from both Gpr39^Ob−/Ob−^ and Gpr39^fl/fl^ mice, and bone marrow was thoroughly flushed out. DNA was then extracted from the remaining bone tissue. The successful deletion of GPR39 in the Gpr39^Ob−/Ob−^ mice was validated using primers that flank exon 1 and provide a signal of 242 base pairs if the exon is deleted. A positive 242 base pair PCR signal was detected in Gpr39^Ob−/Ob−^ but not in Gpr39^fl/fl^. Moreover, the signal from the undeleted allele was weaker in Gpr39^Ob−/Ob−^ compared to Gpr39^fl/fl^ confirming GPR39 deletion at least in part (Figure 1B). Several studies showed that mice expressing Cre recombinase under the control of the osterix promoter (Osx cre) experience alterations in their skeletal structure (Huang and Olsen 2015). Therefore, Osx cre mice were used as an additional control in all experiments. Four‐month‐old female mice harboring osteoblast lineage‐specific deletion of GPR39 (*Gpr39* ^*Ob−/Ob−*^) were both shorter and lighter than *Gpr39* ^*Fl/Fl*^ and Osx cre mice (Figure 3A,B). µCT analysis revealed that *Gpr39* ^*Ob−/Ob−*^ mice had a considerably lower trabecular bone volume fraction compared to both *Gpr39* ^*Fl/Fl*^ and Osx cre mice (Figure 3C). Of note, Osx cre mice had significantly higher bone volume fraction than *Gpr39* ^*Fl/Fl*^ mice. This finding is similar to a previous study that showed that Osx cre mice had a decreased body weight and delayed growth, but eventually overcame these issues as they matured, resulting in slightly higher bone volume than control mice (Davey et al. 2012). Analysis of trabecular architecture showed that *Gpr39* ^*Ob−/Ob−*^ had significantly thinner trabecular compared to the control groups (Figure 3D). Trabecular numbers were similar in the vertebra of *Gpr39* ^*Ob−/Ob−*^ and *Gpr39* ^*Fl/Fl*^ mice and were lower in both groups compared to vertebra of Osx cre mice (Figure 3E). Trabecular separation values were similar in all experimental groups (Figure 3F). Femoral cortical structural analysis showed significant reduction of cortical bone area (Figure 3G) and cortical thickness (Figure 3H) in *Gpr39* ^*Ob−/Ob−*^ mice. However, the percentage of cortical bone area to total tissue area (Figure 3I) was similar in all groups suggesting the reduction in cortical area and thickness was attributed to the smaller size of *Gpr39* ^*Ob−/Ob−*^ bones compared to the control groups (Figure 3A). Cortical BMC was similar in all experimental groups (Figure 3J). These data suggest that GPR39 activity in the osteoblast lineage is important for maintaining proper bone structure.

Subsequent static and dynamic histomorphometric analyses demonstrated that *Gpr39* ^*Ob−/Ob−*^ mice exhibited significantly elevated mineralizing surfaces (Figure 3G,K), mineral apposition rates (Figure 3H,L), and bone formation rates (Figure 3I,M), indicating that GPR39 modulates both the magnitude and tempo of osteoblastic mineral deposition. Quantitative assessment of osteoclastic parameters revealed a marked increase in osteoclast perimeter (Figure 3J,N) and osteoclast number (Figure 3K,O) relative to the controls. Notably, the ratio of serum concentrations of RANKL to OPG remained comparable between *Gpr39* ^*Ob−/Ob−*^and *Gpr39* ^*Fl/Fl*^ mice, with both ratios significantly lower than that observed in Osx‐Cre mice (Figure 1P). These data suggest that the increased osteoclast distribution observed in *Gpr39* ^*Ob−/Ob−*^ mice does not result from elevated systemic levels of circulating RANKL or OPG but rather reflects local regulatory mechanisms governed by GPR39.

### 3.4 GPR39 Activity in the Osteoblast Lineage Is Essential for Proper Collagen Synthesis and Its Deposition

**Figure 4:**
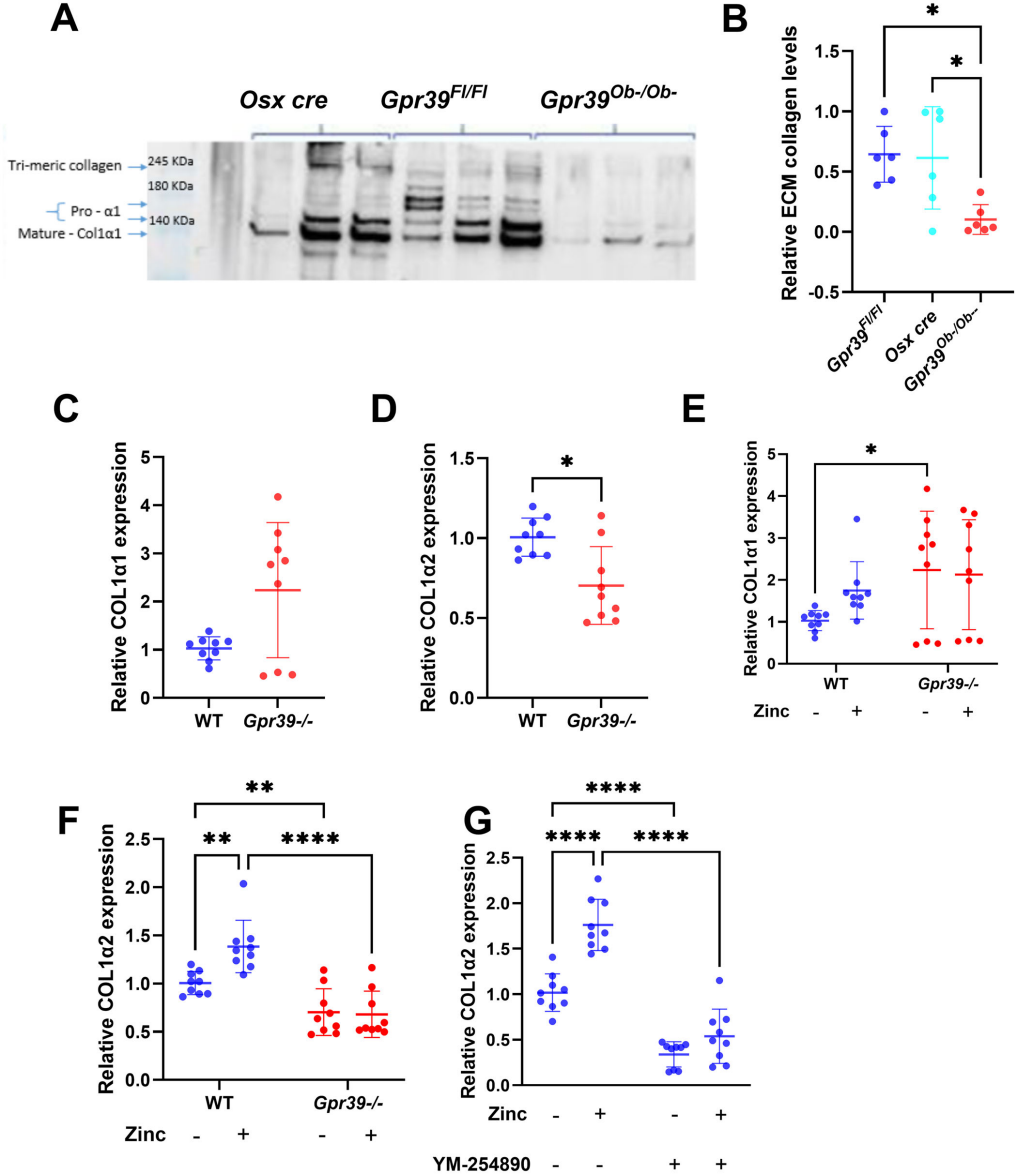
GPR39 regulates collagen expression and deposition in osteoblasts. Primary osteoblasts from the indicated mice were cultured in osteogenic medium. (A) Western blot of extracellular collagen type I alpha I in protein lysates after 21 days of differentiation. Trimeric collagen, partial digested collagen (Pro‐α1), and mature collagen bands are shown. (B) Densitometric analysis of collagen (140 kDa) from three samples across two independent experiments. (C and D) Quantitative analysis of Col1a1 and Col1a2 mRNA in osteoblasts differentiated for 17 days. (E and F) Col1a1 and Col1a2 mRNA levels in osteoblasts from WT and *Gpr39* ^−/−^ mice incubated with (+) or without (−) zinc. (G) Col1a2 mRNA expression in WT osteoblasts treated with zinc, with or without the Gαq inhibitor YM‐254890. All Col1a2 mRNA levels were normalized to actin and B2M, with WT samples without zinc used as a reference for fold change. Statistical significance was assessed using one‐way ANOVA (B–D) and two‐way ANOVA (E–G). *p < 0.05, *﻿﻿*p* < 0.01, ***p < 0.001, and ****p < 0.0001.

To determine whether GPR39 directly influences collagen production in osteoblasts, osteoblasts isolated from *Gpr39* ^*Ob−/Ob−*^ mice were analyzed. Western blotting with a collagen type I‐specific polyclonal antibody which recognize collagen type I alpha I chain revealed significantly reduced extracellular collagen levels in *Gpr39* ^*Ob−/Ob−*^ osteoblasts compared to *Gpr39* ^*Fl/Fl*^ and *Osx‐Cre* osteoblasts, indicating impaired collagen synthesis and deposition due to GPR39 deficiency (Figure 4A,B).

To assess whether GPR39 regulates Col1 mRNA expression, quantitative analysis of Col1a1 and Col1a2 transcripts was performed in differentiating osteoblasts. The analysis revealed significantly reduced Col1a2, but not Col1a1, expression in *Gpr39* ^−/−^ osteoblasts compared to wild‐type (WT) controls (Figure 4C,D). To evaluate whether zinc, a known activator of GPR39, induces Col1 gene expression, we examined the effects of acute zinc exposure (two 1‐min pulses over a 48‐h period) on Col1a1 and Col1a2 expression in WT and *Gpr39* ^−/−^ osteoblasts.

Two‐way ANOVA of Col1a1 expression identified a significant effect of genotype (*p* = 0.0271), whereas the effects of zinc treatment (*p* = 0.3789) and the genotype–zinc interaction (*p* = 0.2318) were not significant (Figure 4E). Post hoc analysis indicated that Col1a1 expression was significantly lower in untreated WT cells compared to untreated Gpr39^−/−^ cells (*p* = 0.0177), while no other group differences were observed. These results suggest that changes in Col1a1 expression are not mediated by zinc signaling through GPR39.

In contrast, analysis of Col1a2 expression revealed a significant interaction between genotype and zinc treatment (*p* = 0.0121), indicating that the zinc response differed between genotypes. Pairwise comparisons demonstrated that zinc significantly upregulated Col1a2 expression only in WT osteoblasts, suggesting that zinc regulates Col1a2 expression in a GPR39‐dependent manner (Figure 4F).

Zinc binding to GPR39 activates Gαq‐mediated signaling, prompting investigation into its role in Col1a2 regulation. Zinc‐induced Col1a2 expression was abolished by the Gαq inhibitor YM‐254890 (*p* = 0.0018), whereas *Gpr39* ^−/−^ osteoblasts remained unresponsive to both zinc and YM‐254890 treatment (Figure 4G). These findings indicate that GPR39 regulates Col1a2 expression through a zinc‐dependent, Gαq‐mediated signaling pathway, potentially accounting for the impaired collagen deposition observed in GPR39‐deficient osteoblasts.

## 4 Discussion

This study provides new insights into the role of the zinc‐sensing G protein‐coupled receptor GPR39 in skeletal homeostasis, with particular emphasis on its sex‐specific effects and osteoblast lineage‐intrinsic function. Building upon earlier evidence demonstrating that GPR39 supports trabecular bone mass and matrix quality in male mice (Jovanovic et al. 2018), the present work expands these findings by characterizing its role in female and ovariectomized (OVX) mice—models pertinent to postmenopausal osteoporosis.

Our findings reveal that GPR39 deficiency leads to a more severe osteoporotic phenotype in female mice compared to males, characterized by a reduction in bone mass, abnormal mineralization, and increased osteoclast distribution. These effects appear to be modulated, at least in part, by estrogen, which is known to attenuate osteoclastogenesis and bone resorption. The more pronounced impact of GPR39 deficiency in females suggests an interaction between estrogen signaling and GPR39 activity in maintaining bone integrity. In OVX mice, which mimic estrogen‐deficient conditions, GPR39‐deficient mice displayed increased susceptibility to bone loss, further underscoring its role in mitigating bone deterioration in postmenopausal‐like states.

The study also demonstrates that the effects of GPR39 deficiency are age‐dependent. While mature GPR39‐deficient female mice exhibited significant bone loss, younger mice showed no difference compared to controls, suggesting that GPR39's regulatory effects on bone become more critical after skeletal maturity. Additionally, deletion of GPR39 using Osx‐Cre, which targets the osteoblast lineage including preosteoblasts, osteoblasts, bone marrow stromal cells, and osteocytes, recapitulates the global knockout phenotype, supporting a cell‐intrinsic role of GPR39 in the osteoblast lineage. Although Osx‐Cre is widely used to drive recombination in osteolineage cells, it is well established that this promoter is not strictly osteoblast‐specific and exhibits activity in bone marrow adipocytes, stromal progenitors, and osteoblast‐derived osteocytes (Rodda and McMahon 2006). Therefore, the term “osteoblast lineage‐specific” is used throughout the manuscript.

Consistent with our previous report (Jovanovic et al. 2018), GPR39 deficiency was associated with impaired collagen synthesis and abnormal mineralization. Gpr39‐deficient osteoblasts exhibited reduced Col1a2 expression and impaired collagen deposition as type I collagen is a heterotrimer composed of two α1(I) and one α2(I) chains, decreased Col1a2 transcription likely impairs proper triple‐helix assembly. This may destabilize unassembled COL1A1 chains, resulting in their increased degradation. This mechanism is supported by studies in Col1a2‐deficient murine models of osteogenesis imperfecta, where abnormal α1(I)_3_ homotrimers exhibit structural instability and are inefficiently incorporated into the extracellular matrix (Roberts‐Pilgrim et al. 2011).

Interestingly, despite stable Col1a1 mRNA expression in Gpr39‐deficient osteoblasts, COL1A1 protein levels were markedly reduced, suggesting posttranscriptional regulatory mechanisms. One plausible explanation is enhanced proteasomal degradation of misfolded or unassembled collagen subunits. Zinc plays a crucial role in maintaining proteostasis, in part through its structural and catalytic functions in E3 ubiquitin ligases and deubiquitinases. For example, the deubiquitinase Rpn11, which is essential for substrate processing at the proteasome, requires zinc for its catalytic activity (Verma et al. 2002). GPR39, as a zinc‐sensing receptor, may regulate these enzymes either directly or indirectly via zinc‐dependent signaling cascades. Disruption of GPR39 function could therefore perturb proteostasis, facilitating increased collagen degradation.

In our prior study, Fourier‐transform infrared spectroscopy revealed a significantly elevated mineral‐to‐matrix ratio in male *Gpr39* ^*−/−*^ mice, attributed to a reduction in the amide I peak—indicative of lower organic matrix content. However, no spectral features consistent with collagen immaturity, such as altered collagen cross‐linking or changes in collagen maturity indices, were observed. These findings indicate that while GPR39 deficiency leads to reduced collagen quantity, there is no histological or spectroscopic evidence of defective collagen maturation. Similarly, the current findings in females show increased cortical BMC and elevated mineralization indices—such as mineralizing surface and mineral apposition rate—yet trabecular bone volume (BV/TV), trabecular thickness (Tb.Th), and number (Tb.N) are significantly reduced.

This apparent paradox, in which dynamic bone formation parameters are increased despite reduced radiographic bone volume, reflects a pathological uncoupling between mineral deposition and matrix production. Rather than indicating enhanced bone accrual, the elevated mineralization represents premature deposition of mineral onto a quantitatively and qualitatively compromised collagen scaffold. This pathological mineralization results in brittle bone architecture, as supported by the reduced osteoid thickness and increased mineral density observed in GPR39‐deficient males (Jovanovic et al. 2018), and by the decreased and variable serum PINP levels in females—suggesting suppressed and dysregulated collagen turnover.

These findings are consistent with clinical contexts in which matrix quality is impaired despite ongoing mineralization. For example, in patients receiving combined anabolic and antiresorptive therapy, serum PINP levels may not accurately reflect bone formation activity due to suppressed collagen dynamics (Greenblatt et al. 2017). Moreover, longitudinal studies such as FLEX and HORIZON have demonstrated that PINP may fail to predict bone formation or bone loss in patients with previously suppressed bone turnover.

Cortical bone analysis revealed a significant reduction in cortical area and thickness in *Gpr39* ^*Ob*−*/Ob*−^ mice, while the cortical bone area relative to total area (Ct.Ar/Tt.Ar) remained unchanged. These data indicate that cortical structural deficits reflect reduced overall bone size rather than impaired mineral accumulation per se. Cortical BMC was not altered in conditional knockouts, in contrast to global Gpr39 knockouts, where BMC was elevated. This phenotypic divergence may be partially attributable to Cre‐driver‐specific effects. Although cortical thickness and area were similar in *Gpr39* ^*Fl/Fl*^ and Osx‐Cre control mice, mean cortical TMD in Osx‐Cre mice was modestly lower (albeit not statistically significant) than in *Gpr39* ^*Fl/Fl*^.

In parallel with matrix‐related abnormalities, a marked increase in osteoclast perimeter and number was detected in Gpr39^−/−^ female mice and in mice with osteoblast lineage‐specific deletion of GPR39, whereas this phenotype was absent in Gpr39^−/−^ male mice. To investigate whether this osteoclastogenic response reflected systemic alterations in osteoclast‐activating cytokines, serum levels of RANKL and OPG were quantified. The RANKL/OPG ratio remained unchanged between GPR39‐deficient and control mice, indicating that the enhanced osteoclast distribution was not driven by systemic shifts in these canonical regulators.

This dissociation between systemic RANKL/OPG levels and local osteoclast activation is consistent with previous reports. For example, conditional deletion of Tnfrsf11b (OPG) in osteoblasts increases bone resorption without altering serum RANKL or OPG concentrations (Cawley et al. 2020). Likewise, in disuse osteoporosis following spinal cord injury, robust bone resorption is not accompanied by temporal changes in circulating RANKL or OPG (Maïmoun et al. 2005). Furthermore, pro‐inflammatory cytokines such as TNF‐α can potentiate osteoclast activation through RANKL‐independent mechanisms, even when RANKL is neutralized by OPG (Fuller et al. 2002). These studies underscore the importance of local cell–cell interactions at the bone surface and support the notion that GPR39 regulates osteoclast activity through local mechanisms, possibly via modulation of membrane‐bound RANKL or osteoblast‐derived clastokines.

It is important to note that GPR39 has been reported to respond to several endogenous ligands, including zinc, bile acids, eicosanoids, and glycoprotein nonmetastatic melanoma protein B (Kaul et al. 2022; Ramadoss et al. 2024; Zi and Rao 2024). Although the present study focused on zinc‐mediated signaling and its downstream consequences in bone cells, it cannot exclude the potential contributions of other GPR39 ligands in vivo. Therefore, while the data provide strong evidence for GPR39‐dependent regulation of osteoblast function, collagen synthesis, and local osteoclast distribution, these findings should be interpreted with caution in light of the receptor's complex ligand profile and potential context‐dependent activation.

In summary, our discoveries underscore the pivotal role of GPR39 in the regulation of bone homeostasis, particularly through its sex‐specific modulation of osteoblast function and collagen synthesis, which collectively highlight its potential as a therapeutic target for preserving bone integrity under conditions of estrogen deficiency.

## Author Contributions

Conceptualization, Noam Levaot; methodology, Biplab Chaterjee, Anton Davydok, Johannes Krug, Chen Abramovitch‐Dahan, and Katharina Jähn‐Rickert; image analysis, Biplab Chaterjee, Gal Gozlan, and Chen Abramovitch‐Dahan; validation, Biplab Chaterjee, Anton Davydok, Johannes Krug, Katharina Jähn‐Rickert, Noam Levaot, and Chen Abramovitch‐Dahan; formal analysis, Anat Reiner‐Benaim; investigation, Biplab Chaterjee, Gal Gozlan, Anton Davydok, Johannes Krug, Chen Abramovitch‐Dahan, and Katharina Jähn‐Rickert; resources, Björn Busse and Noam Levaot; writing – original draft preparation, Biplab Chaterjee, Chen Abramovitch‐Dahan, and Noam Levaot; writing – review and editing, Gal Gozlan, Chen Abramovitch‐Dahan, Katharina Jähn‐Rickert, Björn Busse, Anat Reiner‐Benaim, and Noam Levaot; visualization, Gal Gozlan, Noam Levaot and Chen Abramovitch‐Dahan; supervision, Chen Abramovitch‐Dahan, Katharina Jähn‐Rickert, Björn Busse, and Noam Levaot; funding acquisition, Björn Busse and Noam Levaot. All authors have read and agreed to the published version of the manuscript.

## Conflicts of Interest

The authors declare no conflicts of interest.

## Supporting information


**Supplemental Figure 1:** Validation of GPR39 exon 1 deletion in bone tissue. DNA was extracted from tibiae of mice following complete removal of bone marrow. (Upper panel): Schematic representation of the GPR39 locus, illustrating the floxed exon 1 configuration in wild‐type (WT) alleles (*GPR39* ^*FL/FL*^) and the excised allele (*GPR39* ^*Ob*−*/Ob*−^) following Cre‐mediated recombination in Osx‐Cre transgenic mice. LoxP sites are indicated by blue arrowheads. Yellow arrows denote PCR primers designed to amplify the floxed allele prior to recombination, while black arrows indicate primers used to detect the excised allele post‐recombination. (Lower panel): Representative gel electrophoresis image demonstrating the presence of a 242 bp PCR product, specifically marking the deleted allele in bone tissue of GPR39^*Ob*−*/Ob*−^ mice.

## Data Availability

The data presented in this study are available on request from the corresponding author.
