# Supplementary material for: The Role of GPR39 in Regulating Osteoblast Function, Bone Matrix Quality, and Gender‐Specific Bone Homeostasis
Source: J Cell Physiol. 2025 Oct 3;240(10):e70095. doi: 10.1002/jcp.70095 (PMC12492777; doi:10.1002/jcp.70095)

Supplemental Figure 1. Validation of GPR39 exon 1 deletion in bone tissue. DNA was extracted from tibiae of mice following complete removal of bone marrow. (Upper panel): Schematic representation of the GPR39 locus, illustrating the floxed exon 1 configuration in wild-type (WT) alleles (*GPR39^FL/FL^*) and the excised allele (*GPR39^Ob-/Ob-^*) following Cre-mediated recombination in Osx-Cre transgenic mice. LoxP sites are indicated by blue arrowheads. Yellow arrows denote PCR primers designed to amplify the floxed allele prior to recombination, while black arrows indicate primers used to detect the excised allele post-recombination. (Lower panel): Representative gel electrophoresis image demonstrating the presence of a 242 bp PCR product, specifically marking the deleted allele in bone tissue of GPR39^Ob-/Ob- mice.


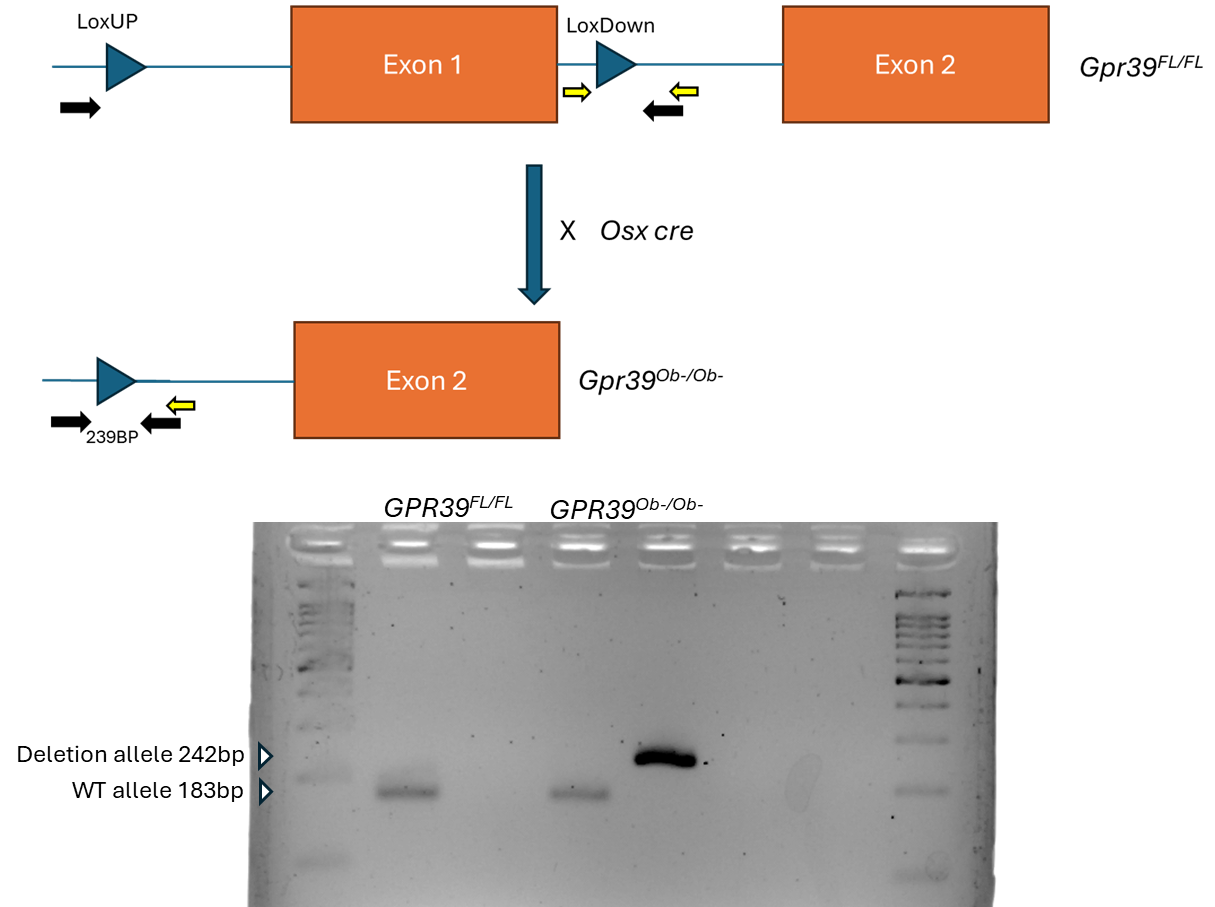

Supplement: Supplementary file 1 — Supplemental Figure 1: Validation of GPR39 exon 1 deletion in bone tissue. DNA was extracted from tibiae of mice following complete removal of bone marrow. (Upper panel): Schematic representation of the GPR39 locus, illustrating the floxed exon 1 configuration in wild‐type (WT) alleles (GPR39 FL/FL ) and the excised allele (GPR39 Ob−/Ob−) following Cre‐mediated recombination in Osx‐Cre transgenic mice. LoxP sites are indicated by blue arrowheads. Yellow arrows denote PCR primers designed to amplify the floxed allele prior to recombination, while black arrows indicate primers used to detect the excised allele post‐recombination. (Lower panel): Representative gel electrophoresis image demonstrating the presence of a 242 bp PCR product, specifically marking the deleted allele in bone tissue of GPR39 Ob−/Ob− mice. [file JCP-240-0-s001.docx]
